# Evaluation on coupling coordinated development of population economy and eco-geological environment in the twin-city economic circle of Chengdu–Chongqing region

**DOI:** 10.1038/s41598-023-40352-w

**Published:** 2023-08-18

**Authors:** Dong Han, Liang Chen, Hao Wu, Xinyuan Wang, Yi Xiao, Haonan Yang, Shiyu Liu, Shuangshuang Xu, Huan Huang, Ming Chang

**Affiliations:** 1 College of Management Science, Chengdu University of Technology, Chengdu, 610059 Sichuan China; 2 School of Finance and Accounting, Chengdu Jincheng College, Chengdu, 610097 Sichuan China; 3 Geomathematics Key Laboratory of Sichuan Province, Chengdu University of Technology, Chengdu, 610059 Sichuan China; 4 College of Business, Chengdu University of Technology, Chengdu, 610059 Sichuan China; 5 Digital Hu Huanyong Line Research Institute, Chengdu University of Technology, Chengdu, 610059 Sichuan China; 6 Key Laboratory of Digital Earth Science, Aerospace Information Research Institute, Chinese Academy of Sciences, Beijing, 100094 China; 7 State Key Laboratory of GeoHazard Prevention and GeoEnvironment Protection, Chengdu University of Technology, Chengdu, 610059 Sichuan China

**Keywords:** Environmental sciences, Environmental social sciences

## Abstract

Using the CRITIC method, comprehensive index evaluation method, VAR model, coupling coordination model and other methods, this paper evaluates the comprehensive development of the population economy and eco-geological environment composite system in the twin-city economic circle of Chengdu–Chongqing region from 2000 to 2020, verifies the dynamic coupling relationship between subsystems and measures the coupling coordination degree of complex system. Meanwhile, the differences in the development process of each subsystem are discussed and the spatial–temporal evolution characteristics and laws of coupling coordination of the composite system during the research period are analyzed. The results reveal the following: (1) There is a long-term coordinated relationship between population, economy and eco-geological environment in Chengdu–Chongqing region, which have the conditions for emergence and generation. (2) The subsystems of population, economy and eco-geological environment in Chengdu–Chongqing region show an overall upward trend, among which the Sichuan part obviously outperforms the Chongqing part. Besides, the growth rate of the economy subsystem is significantly higher than that of the population, eco-geological environment subsystem. (3) The coupling coordinated development of the composite system has shown a benign upward development trend, gradually changing from “composite spiral structure” to “two cores outstanding, peripheral collapse, the west superior to the east”, while the main coordination state has developed from “Basic synergy—Economic lag” in 2000 to “Basic synergy—Population lag” in 2022. In addition, Cheng-De-Mian and Yuzhong districts with the better level of economic development have shown “Advanced synergy—Ecological lag”.

## Introduction

In October 2020, General Secretary Xi Jinping presided over the Political Bureau of the Communist Party of China (CPC) Central Committee meeting to review the *Outline of the Construction Plan of Twin-city economic circle in Chengdu–Chongqing region*, which has established the strategic positioning of the construction of the Chengdu–Chongqing Twin City economic Circle as “an important economic center with national influence, a center of scientific and technological innovation, a new highland of reform and opening up, a livable place for quality life, an important source of growth and new power for promoting high-quality national development”. Chengdu–Chongqing region is both an economic growth pole and a densely populated area in the central and western regions of China, and is also rich in natural and human resources, whose periphery is not only an ecological barrier zone and a water protection area, but also a geologically fragile and environmentally sensitive area. According to the above background, it is undoubtedly a systematic and complicated problem to study the coordinated development of human-land relationship in Chengdu–Chongqing region, which involves regional population, economy, resources, ecology and environment. Therefore, the research on the security of ecological geological environment in Chengdu–Chongqing region should be carried out, while exploring and evaluating the sustainable interactions between regional population economy and ecological geological environment, the law of cooperative evolution, and building a collaborative driving mechanism, which are not only the local practice of "Chinese-style modernization is the modernization of harmonious coexistence of human and nature" proposed by the 20th National Congress of the Communist Party of China, but also contribute to the realization of "high standards, high quality, promoting the construction of Chengdu–Chongqing twin cities economic circle".

At present, the relevant research of domestic and international scholars has mainly conducted numerous studies on the following aspects: First, the research dimension focuses on the correlation analysis of population, economy and environment. From population and economy^1^, economy and environment^2–6^, population and environment^7,8^, and among them^9^, analysis of their coordination relationship; Second, the characteristics of wide scope and multiple levels on the spatial scale are presented, such as national level^10,11^, watershed level^12^, provincial level^13,14^, and a few scholars have conducted similar research on the twin-city economic circle in Chengdu–Chongqing region^15^; Third, the research method has gradually shifted from qualitative to quantitative analysis, while quantitative research has been carried out through mathematical modeling methods such as coupling coordination model, and grey relation, etc.

To sum up, there is currently a relative lack of relevant research on the coordinated development of population economy and ecological environment in Chengdu–Chongqing twin-city economic circle, especially which consider the impact of geographical and geological environment on economy and society in Chengdu–Chongqing region. On this basis, this paper intends to construct an evaluation index system for the coordinated development of population economy and eco-geological environment, while the panel data and remote sensing data are processed by using the CRITIC method^16^, comprehensive index evaluation method, VAR model and coupling coordination model in order to analyze the spatial–temporal evolution characteristics and laws of coupling coordination relationship in the compound system which consists of population, economy and eco-geological environment systems in Chengdu–Chongqing Economic circle from 2000 to 2020. Furthermore, this paper makes research on the evolution mode of its subsystem development state in order to provide decision-making basis for the sustainable development of population economy and eco-geological environment in the study area.

## Research area and research methods

### Overview of the research area

**Figure 1:**
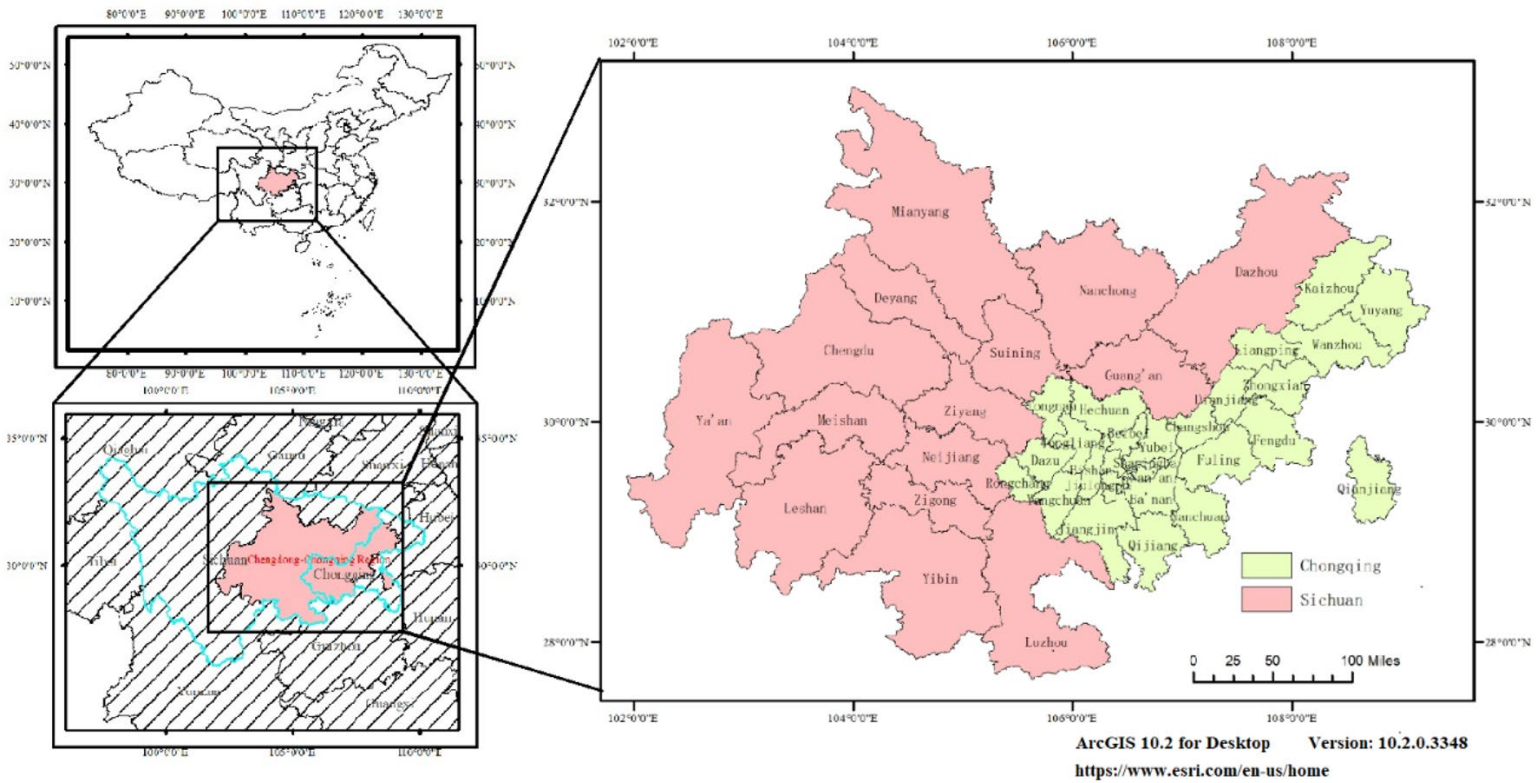
Overview of Chengdu–Chongqing Region’s Geographical Location and Administrative Division.

Twin-city economic circle in Chengdu–Chongqing region (hereinafter referred to as “Chengdu–Chongqing region”) is located in the upper reaches of the Yangtze River and the Sichuan Basin, where the Belt and Road initiative meets the Yangtze River Economic Belt, and which borders Hunan Province and Hubei Province to the east, Qinghai Province and Tibet Autonomous Region to the west, Yunnan Province and Guizhou Province to the south and Shaanxi Province and Gansu Province to the north, with a total area of 18.5 × 10^4^ km^2^ (Fig. 1) . Based on the availability of data, this paper takes Mianyang, Dazhou, Ya’an, Kaizhou, Yunyang and other areas as a whole into the scope of the study area on the basis of the areas delineated in the *Outline of the Construction Plan of Twin-city economic circle in Chengdu–Chongqing region* issued by the State Council, the Central Committee of the Communist Party of China (CPC). And in the process of research, 29 districts (counties) in Chongqing Municipality and 15 prefecture-level cities in Sichuan Province are taken as one unit, totaling 44 research units. Aside from this, taking into account the special administrative organization of Chongqing Municipality, Sichuan and Chongqing part are measured separately in the assessment process. Both Figs. 1 and 8 were created by ArcGIS 10.2 for Desktop (Version:10.2.0.3348 https://www.esri.com/en-us/home), based on the Vector Border Map of China’s County level Administrative Division in 2021 (Geographic Coordinate System: CGCS_2000).

### Research methods

#### Index selection

Based on the United Nations human development index, this paper puts forward the population sustainability index (PSI), which comprehensively evaluates the status of the population subsystem from seven aspects: scale, growth, age, education, health, income and residence. As for the economy subsystem, on the basis of the connotation of high-quality economic growth proposed by the Central Committee of the Communist Party of China (CPC), the economic high-quality index (EHI) is put forward, which comprehensively measures the state of economic subsystems from six dimensions: growth, innovation, coordination, green, openness and sharing. Meanwhile, the eco-geological environment background index (EEBI) is proposed to measure the state of the eco-geological environment subsystem from five dimensions: resource elements, ecological elements, ecological pressure, ecological response and geo-geological environment. Depending on the availability and usefulness of data, the indicators contained in different measurement levels have changed. For example, in the verification of the overall dynamic coupling relationship in Chengdu–Chongqing region, the geo-geological environment with little change is not selected, and when coupling coordination measures are adopted, the ecological response index data of Chongqing district (county) is difficult to obtain and cannot be selected. The index system adopted in this paper is shown in Table 1.

**Table 1:** Index system of Coordinated Development of Population Economy and Eco-geological Environment in the twin-city economic circle of Chengdu–Chongqing region.

Primary index	Secondary index	Three-level index	Units (Contents if necessary)	Index orientation	Data source	Use dimension
Population sustainability index (PSI)	Scale	Population density	Person/km^2^	Positive	Census	All
	Residence	Urbanization rate	%	Positive	Census	All
	Growth	Natural growth rate	‰	Positive	Census	Verification
	Age	Labor population rate	%	Positive	Census	All
		Aging rate	%	Negative	Census	Coordinate
	Education	Illiteracy rate	%	Negative	Census	Coordinate
		higher education rate	%	Positive	Census	All
	Health	Number of beds	pcs/10,000 persons	Positive	Statistics	All
	Income	Per capita resident income	RMB/person	Positive	Statistics	Verification
Economic high-quality index (EHI)	Growth	Per capita GDP	Reduction based on 1978, RMB/person	Positive	Statistics	All
		Urban disposable income	RMB/person	Positive	Statistics	Coordinate
		Rural net income	RMB/person	Positive	Statistics	
		Per capita fixed investment	RMB/person	Positive	Statistics	
		Per capita consumption	RMB/person	Positive	Statistics	
		Per capita financial income	RMB/person	Positive	Statistics	
	Innovation	R&D expenditure	R&D Expenditure/GDP, %	Positive	Statistics	Verification
	Coordination	Proportion of secondary industry	Secondary industry GDP/total GDP,%	Positive	Statistics	Coordinate
		Proportion of tertiary industry	GDP of tertiary industry/total GDP,%	Positive	Statistics	All
	Green	Energy consumption level	Energy consumption of ten thousand RMB GDP, tons of standard coal	Negative	Statistics	Coordinate
		Water consumption level	Water consumption of ten thousand RMB GDP, tons	Negative	Statistics	
	Openness	Import and export of goods	Total import and export of goods as a percentage of GDP,%	Positive	Statistics	Verification
		Utilization of foreign capital	Share of FDI in GDP,%	Positive	Statistics	
	Sharing	Per capita fiscal expenditure	RMB/person	Positive	Statistics	All
Eco-geological environment background index (EEBI)	Resource elements	Per capita cultivated land	ha/person	Positive	Statistics	All
		Per capita water resources	m^3^/person	Positive	Statistics	All
		Proportion of plain area	Slope < 3 area/area,%	Positive	Remote sensing	Coordinate
		Agricultural water and soil matching	Agricultural irrigation land area per capita, ha/person	Positive	Statistics	Verification
	Ecological elements	Ecological land	Nature reserve area/land area,%	Positive	Statistics	Verification
		Water area proportion	Water area/land area,%	Positive	Remote sensing	Coordinate
		Vegetation coverage	NDVI mean	Positive	Remote sensing	All
		Forest coverage	Forest area/land area,%	Positive	Statistics	Verification
		Annual average temperature	Average annual temperature,℃	Positive	Statistics	Coordinate
		Average annual rainfall	Average annual rainfall, mm	Positive	Statistics	Coordinate
	Ecological pressure	PM2.5 concentration	Average annual PM2.5 concentration, ug/m^3^	Negative	Statistics	Coordinate
		Waste water discharge	Waste water discharge/GDP, ton/ten thousand RMB	Negative	Statistics	All
		Exhaust emission	Exhaust emissions/GDP, tons/ten thousand RMB	Negative	Statistics	All
		Solid waste generation	General industrial solid waste generation/secondary GDP, ton/ten thousand RMB	Negative	Statistics	All
	Ecological response	Waste utilization	Comprehensive utilization rate of general industrial wastes,%	Positive	Statistics	Verification
		Pollution control/treatment	Industrial pollution control expenses as a percentage of secondary GDP,%	Positive	Statistics	Verification
	Geo-geological environment	Hypsography	Difference between high and low elevation values in the region, m	Negative	Remote sensing	Coordinate
		Elevation	Median elevation in the area, m	Negative	Remote sensing	Coordinate
		Ground motion magnitude	Average absolute value of peak acceleration of regional ground motion, g	Negative	Statistics	Coordinate
		Equal density fault zone	The sum of the lengths of fault structures in the grid unit of fault zone	Negative	Statistics	Coordinate

Based on the availability and usefulness of the data, this paper adopts a differentiated index system for the analysis of the coupling coordination of 44 research and the overall dynamic coupling relationship units in Chengdu–Chongqing area, characterized by the column of “Use Dimension”, in which “Verification” means that the index is used to verify the dynamic coupling relationship, “Coordination” refers to the index used for coupling coordination measure, and “All” stands for the index used in both dimensions of research. Among data sources, “Census” means that data comes from census data, “Statistics” means that data comes from various statistical yearbooks and professional databases, and “Remote sensing” means that data comes from the interpretation and processing of remote sensing images.

#### Data sources

The data of all indicators in this study are mainly derived from Sichuan Provincial Statistical Yearbook (2001–2021), Chongqing Municipal Statistical Yearbook (2001–2021), Statistical Yearbook of Prefecture-level Cities in Sichuan Province and Chongqing District (County), China Environmental Statistics Yearbook, Sichuan and Chongqing Environmental Statistics Yearbooks, while dealing with the data of the missing year by interpolation fitting method. At the meantime, some basic data of eco-geological environment system are mainly obtained by interpreting of LANDSAT 7-ETM and LANDSAT 8-OLI images (spatial resolution: 30 m) and from the website of Resources and Environmental Science Data Center of China Academy of Sciences.

#### Research methods

1. The CRITIC weight method is used to determine the weights of various indicators under the subsystems of population, economy and ecological geological environment^17^. The specific steps are as follows: First, the standard deviation and correlation coefficient of each element are calculated to reflect the variability and correlation of each element in the comprehensive measurement of the population economy and eco-geological environment system.
  1 $$ S_{j} = \sqrt {\frac{{\mathop \sum \nolimits_{i = 1}^{n} (x_{ij} - \overline{x}_{j} )^{2} }}{n - 1}} $$
  2 $${R}_{j}={\sum }_{i=1}^{n}(1-{r}_{ij})$$
  Then, the weights of the elements are determined based on the calculation results (where n represents the number of research objects and m stands for the number of indicators).
  3 $${w}_{j}=\frac{{S}_{j}{R}_{j}}{{\sum }_{j=1}^{m}{S}_{j}{R}_{j}}$$
  Finally, figure out the weights of population, economy and eco-geological environment subsystems in Sichuan and Chongqing, while the development level of each subsystem is evaluated by comprehensive index evaluation method.
  4 $${X}_{j}={\sum }_{j=1}^{n}{w}_{j}{x{\prime}}_{ij}$$
  In the formula, ${X}_{j}$ stands for the comprehensive evaluation of a certain system; ${w}_{j}$ represents the index weight of the $j$ item; ${x\mathrm{^{\prime}}}_{ij}$ is the normalized sample value.
2. The VAR model is used to analyze the dynamic coupling relationship among population, economy and eco-geological environment in Chengdu–Chongqing region^18^. The VAR model regards each endogenous variable in the system as the lag term constructor of all endogenous variables in the system, which can reflect the interactive influence and dynamic correlation between the variables in the system, and with whose advantages, there is no need to set any assumptions and the need for structural models will be avoided, which is suitable for studying the interactive relationship between population, economy and eco-geological environment.
3. The coupling coordination model is used to measure the coordination level of interaction in the population, economy and eco-geological environment system in Chengdu–Chongqing region. The numbers $s_{1} , \ldots ,s_{p}$ are set as the comprehensive evaluation indexes of the subsystems of population, economy, eco-geological environment and other subsystems respectively, which should comply with ${s}_{k}\in (\mathrm{0,1}) (k=\mathrm{1,2},\dots \dots ,\mathrm{p})$. Then the coupling degree model is constructed as follows.
  5 $$C=C({s}_{1},\cdots ,{s}_{p})={\left(\frac{{\prod }_{k=1}^{p}{s}_{p}}{{\left(\frac{1}{p}{\sum }_{k=1}^{p}{s}_{p}\right)}^{p}}\right)}^\frac{1}{p}$$

Then, further construct the coupling coordination model as follows.

6 $${Z}_{j,l,k}=\sqrt{{C}_{j,l,k}\cdot {D}_{j,l,k}}$$

7 $${D}_{j,l,k}=\alpha {X}_{j}+\beta {X}_{l}+\gamma {X}_{k}$$

Drawing on the existing research and considering that population, economy and ecological geological environment are equally important to the development of Chengdu–Chongqing region, this paper sets up $\alpha =\beta =\gamma =1/3$. According to the same gradient, the coupling coordination degree is divided into three categories, five grades and ten grades, which correspond to the coordination level and synergistic grade respectively, as shown in Table 2.

**Table 2:** Classification standard of coupling-coordinated development of population economy and eco-geological environment system.

Stage	Coupling degree	Coordination level	Synergistic grade
Coordinate	0.9000–1.0000	High-quality coordinated development	Advanced synergy
	0.8000–0.8999	Well coordinated development	
	0.7000–0.7999	Intermediate coordinated development	Primary synergy
	0.6000–0.6999	Primary coordinated development	
Transition	0.5000–0.5999	Reluctantly coordinated development	Basic synergy
	0.4000–0.4999	On the verge of imbalance antagonism	
Antagonism	0.3000–0.3999	Mild maladjustment antagonism	Basic maladjustment
	0.2000–0.2999	Moderate imbalance antagonism	
	0.1000–0.1999	Severe imbalance antagonism	Serious maladjustment
	0.0000–0.0999	Extreme imbalance antagonism	

## Analysis of dynamic coupling relationship

### Development status of population economy and eco-geological environment system in Chengdu–Chongqing region

**Figure 2:**
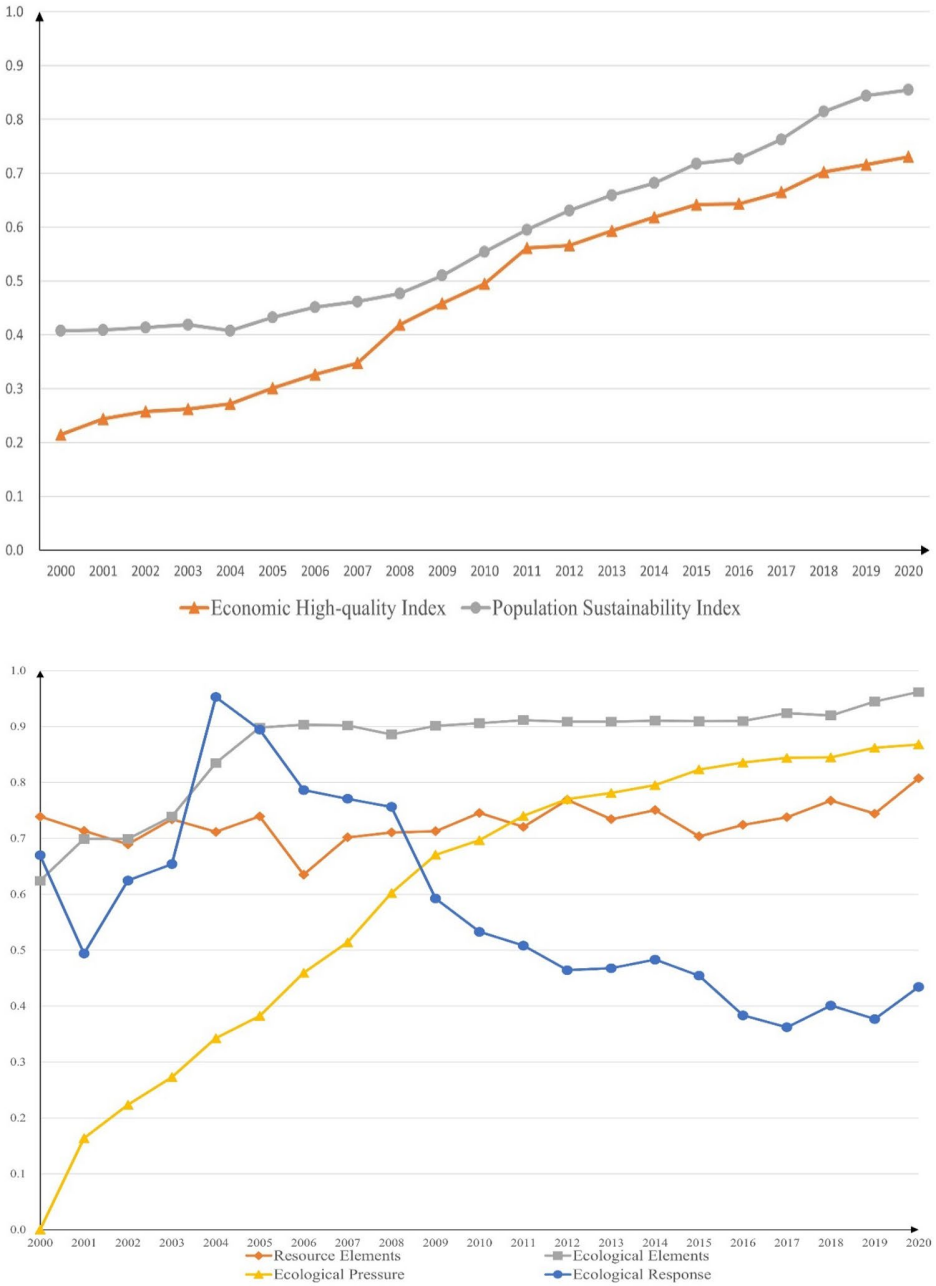
PSI, EHI and EEBI developed in Chengdu–Chongqing region from 2000 to 2020.

Based on VAR model, this paper analyzes the dynamic coupling relationship in the system of population, economy and eco-geological environment between Chengdu and Chongqing, which verifies the existing long-term equilibrium relationship and tests the influence and feedback mechanism formed by the coupling. According to the verification indicators in Table 1, the PSI, EHI and EEBI of Chengdu–Chongqing region from 2000 to 2020 can be obtained, as shown in Fig. 2.

We can see that PSI and EHI of the Chengdu–Chongqing region from 2000 to 2020 maintained an overall upward trend except for occasional years. Meanwhile, among the four dimensions of EEBI, the resource elements in the region have fluctuated slightly, while the ecological elements have rapidly increased and remained stable at high levels. The ecological pressure has been rapidly improved, and correspondingly, after the resource elements, ecological elements, and ecological pressure are relatively stable, the ecological response gradually decreases.

### VAR model construction of population, economy and ecological and geological environment in Chengdu–Chongqing region

From the validation results of the VAR model, there is a stable long-term cointegration relationship between population, economy and ecological and geological environment in the Chengdu–Chongqing region. Due to the limitation of space, the details and graphs of the VAR model construction are not listed in detail in this paper. The validation results of each step are as follows:

#### ADF unit root test

The 5 time series of population (LNX1), economy (LNX2), resource elements (LNY1), ecological elements (LNY2) and ecological pressure (LNY3) are smooth, and ecological response (LNY4) becomes a smooth series after first-order difference. Since DLNY4 is of realistic significance and characterizes the change in the government’s ecological response input, this paper uses the change in ecological response of DLNY4 to characterize the ecological response.

#### Optimal lag order

The nine VAR models composed of four sub-indicators of regional population sustainability, high economic quality and ecological and geological background with the best lag orders of 1, 4, 4, 1, 4, 4, 4, 4, 4, 4, 2, respectively.

####  Endogeneity test

①The causal relationship between population and economy. The variable $\mathrm{ln}{x}_{2}$ is an endogenous variable of $\mathrm{ln}{x}_{1}$, and the opposite is not true, high-quality economic development is the motive force of sustainable population development. ②The causal relationship between population and ecological and geological environment. The variable $\mathrm{ln}{x}_{1}$ is an endogenous variable of $\mathrm{ln}{x}_{1}$, and the opposite does not hold, sustainable population development is the driver of resource factors; the variables $\mathrm{ln}{x}_{1}$ and $\mathrm{ln}{x}_{2}$ are mutually endogenous variables, sustainable population development and ecological factors are mutual causes; the variable $\mathrm{ln}{x}_{1}$ is an endogenous variable of $\mathrm{ln}{x}_{3}$, and the opposite does not hold, sustainable population development is the driver of ecological pressure; the variable D $\mathrm{ln}{x}_{4}$ is endogenous variable of $\mathrm{ln}{x}_{1}$, and vice versa does not hold, sustainable population development is influenced by changes in ecological response. ③The relationship between economy and ecological geological environment. The variables $\mathrm{ln}{x}_{2}$ and $\mathrm{ln}{x}_{1}$ are endogenous to each other ($\mathrm{ln}{x}_{1}$ is within 0.1 significance range), economic quality development and resource factors influence each other; variable $\mathrm{ln}{x}_{2}$ is an endogenous variable of $\mathrm{ln}{x}_{2}$, and the opposite does not hold, economic quality development is influenced by ecological factors; variable $\mathrm{ln}{x}_{2}$ is an endogenous variable of $\mathrm{ln}{x}_{3}$, and the opposite does not hold, economic quality development will act The variable $\mathrm{ln}{x}_{2}$ is an endogenous variable of D $\mathrm{ln}{x}_{4}$, and the opposite does not hold, economic quality development will affect the change of ecological response. Based on practical experience, the above explanations are realistic.

#### Stability test

All the roots and modes of the 9 VAR models in 5 groups are less than 1, which are stable and the various tests done for this VAR model are also valid.

#### VAR modeling

The results of the VAR model fitting are as follows

8 $$ \left[ {\begin{array}{*{20}c} {\ln x_{1} } \\ {\ln x_{2} } \\ \end{array} } \right] = \left[ {\begin{array}{*{20}c} {0.7574} & {0.1750} \\ { - 0.1904} & {1.065} \\ \end{array} } \right]\left[ {\begin{array}{*{20}c} {\ln x_{1,t - 1} } \\ {\ln x_{2,t - 1} } \\ \end{array} } \right] + \left[ {\begin{array}{*{20}c} {0.0395} \\ {0.0020} \\ \end{array} } \right] $$

9 $$ \begin{aligned} \left[ {\begin{array}{*{20}c} {\ln y_{1} } \\ {\ln x_{2} } \\ \end{array} } \right] = & \left[ {\begin{array}{*{20}c} { - 0.0{\kern 1pt} 681} & { - 0.7693} \\ { - 0.1617} & {1.0325} \\ \end{array} } \right]\left[ {\begin{array}{*{20}c} {\ln y_{1,t - 1} } \\ {\ln x_{1,t - 1} } \\ \end{array} } \right] + \left[ {\begin{array}{*{20}c} {0.1181} & {1.7359} \\ {0.0027} & { - 0.1390} \\ \end{array} } \right]\left[ {\begin{array}{*{20}c} {\ln y_{1,t - 2} } \\ {\ln x_{1,t - 2} } \\ \end{array} } \right] \\ & + \left[ {\begin{array}{*{20}c} { - 0.5434} & { - 0.5783} \\ { - 0.2975} & {0.5871} \\ \end{array} } \right]\left[ {\begin{array}{*{20}c} {\ln y_{1,t - 3} } \\ {\ln x_{1,t - 3} } \\ \end{array} } \right] + \left[ {\begin{array}{*{20}c} { - 0.3828} & { - 0.2320} \\ { - 0.4342} & { - 0.4401} \\ \end{array} } \right]\left[ {\begin{array}{*{20}c} {\ln y_{1,t - 4} } \\ {\ln x_{1,t - 4} } \\ \end{array} } \right] + \left[ {\begin{array}{*{20}c} { - 0.5221} \\ { - 0.2384} \\ \end{array} } \right] \\ \end{aligned} $$

10 $$ \begin{aligned} \left[ {\begin{array}{*{20}c} {\ln y_{2} } \\ {\ln x_{1} } \\ \end{array} } \right] = & \left[ {\begin{array}{*{20}c} {0.6091} & {0.0356} \\ {0.3123} & {0.9762} \\ \end{array} } \right]\left[ {\begin{array}{*{20}c} {\ln y_{2,t - 1} } \\ {\ln x_{1,t - 1} } \\ \end{array} } \right] + \left[ {\begin{array}{*{20}c} { - 0.2154} & {0.2300} \\ { - 0.1684} & { - 0.1310} \\ \end{array} } \right]\left[ {\begin{array}{*{20}c} {\ln y_{2,t - 2} } \\ {\ln x_{1,t - 2} } \\ \end{array} } \right] \\ & + \left[ {\begin{array}{*{20}c} { - 0.1248} & { - 0.4299} \\ { - 0.3460} & { - 0.1169} \\ \end{array} } \right]\left[ {\begin{array}{*{20}c} {\ln y_{2,t - 3} } \\ {\ln x_{1,t - 3} } \\ \end{array} } \right] + \left[ {\begin{array}{*{20}c} {0.1214} & {0.2848} \\ {0.5246} & {0.1736} \\ \end{array} } \right]\left[ {\begin{array}{*{20}c} {\ln y_{2,t - 4} } \\ {\ln x_{1,t - 4} } \\ \end{array} } \right] + \left[ {\begin{array}{*{20}c} { - 0.0190} \\ {0.0456} \\ \end{array} } \right] \\ \end{aligned} $$

11 $$ \left[ {\begin{array}{*{20}c} {\ln y_{3} } \\ {\ln x_{1} } \\ \end{array} } \right] = \left[ {\begin{array}{*{20}c} {0.8478} & { - 0.0406} \\ { - 0.0655} & {0.9060} \\ \end{array} } \right]\left[ {\begin{array}{*{20}c} {\ln y_{3,t - 1} } \\ {\ln x_{1,t - 1} } \\ \end{array} } \right] + \left[ {\begin{array}{*{20}c} { - 0.0259} \\ {0.0231} \\ \end{array} } \right] $$

12 $$ \begin{aligned} \left[ {\begin{array}{*{20}c} {\ln y_{4} } \\ {\ln x_{1} } \\ \end{array} } \right] = & \left[ {\begin{array}{*{20}c} {0.1708} & {0.8765} \\ { - 0.0631} & {0.8326} \\ \end{array} } \right]\left[ {\begin{array}{*{20}c} {\ln y_{4,t - 1} } \\ {\ln x_{1,t - 1} } \\ \end{array} } \right] + \left[ {\begin{array}{*{20}c} { - 0.0255} & { - 0.0614} \\ { - 0.1073} & { - 0.3930} \\ \end{array} } \right]\left[ {\begin{array}{*{20}c} {\ln y_{4,t - 2} } \\ {\ln x_{1,t - 2} } \\ \end{array} } \right] \\ & + \left[ {\begin{array}{*{20}c} {0.5525} & {1.2645} \\ { - 0.1147} & {0.2674} \\ \end{array} } \right]\left[ {\begin{array}{*{20}c} {\ln y_{4,t - 3} } \\ {\ln x_{1,t - 3} } \\ \end{array} } \right] + \left[ {\begin{array}{*{20}c} {0.1483} & {1.8183} \\ { - 0.0743} & {0.2356} \\ \end{array} } \right]\left[ {\begin{array}{*{20}c} {\ln y_{4,t - 4} } \\ {\ln x_{1,t - 4} } \\ \end{array} } \right] + \left[ {\begin{array}{*{20}c} { - 0.01247} \\ {0.0416} \\ \end{array} } \right] \\ \end{aligned} $$

13 $$ \begin{aligned} \left[ {\begin{array}{*{20}c} {\ln y_{1} } \\ {\ln x_{2} } \\ \end{array} } \right] = & \left[ {\begin{array}{*{20}c} { - 0.3249} & { - 0.6493} \\ {0.3349} & {0.9987} \\ \end{array} } \right]\left[ {\begin{array}{*{20}c} {\ln y_{1,t - 1} } \\ {\ln x_{2,t - 1} } \\ \end{array} } \right] + \left[ {\begin{array}{*{20}c} {0.1244} & {0.6915} \\ { - 0.6516} & { - 0.4129} \\ \end{array} } \right]\left[ {\begin{array}{*{20}c} {\ln y_{1,t - 2} } \\ {\ln x_{2,t - 2} } \\ \end{array} } \right] \\ & + \left[ {\begin{array}{*{20}c} { - 0.8235} & {0.2967} \\ { - 0.9595} & {0.7277} \\ \end{array} } \right]\left[ {\begin{array}{*{20}c} {\ln y_{1,t - 3} } \\ {\ln x_{2,t - 3} } \\ \end{array} } \right] + \left[ {\begin{array}{*{20}c} { - 0.3882} & { - 0.2178} \\ { - 0.5605} & { - 0.3100} \\ \end{array} } \right]\left[ {\begin{array}{*{20}c} {\ln y_{1,t - 4} } \\ {\ln x_{2,t - 4} } \\ \end{array} } \right] + \left[ {\begin{array}{*{20}c} { - 0.6534} \\ { - 0.4456} \\ \end{array} } \right] \\ \end{aligned} $$

14 $$ \begin{aligned} \left[ {\begin{array}{*{20}c} {\ln y_{2} } \\ {\ln x_{2} } \\ \end{array} } \right] = & \left[ {\begin{array}{*{20}c} {0.7014} & {0.1197} \\ {0.4232} & {0.2902} \\ \end{array} } \right]\left[ {\begin{array}{*{20}c} {\ln y_{2,t - 1} } \\ {\ln x_{2,t - 1} } \\ \end{array} } \right] + \left[ {\begin{array}{*{20}c} { - 0.3469} & { - 0.0462} \\ { - 0.1192} & {0.1162} \\ \end{array} } \right]\left[ {\begin{array}{*{20}c} {\ln y_{2,t - 2} } \\ {\ln x_{2,t - 2} } \\ \end{array} } \right] \\ & + \left[ {\begin{array}{*{20}c} {0.02325} & { - 0.0489} \\ {0.1557} & {0.4433} \\ \end{array} } \right]\left[ {\begin{array}{*{20}c} {\ln y_{2,t - 3} } \\ {\ln x_{2,t - 3} } \\ \end{array} } \right] + \left[ {\begin{array}{*{20}c} { - 0.0511} & {0.0324} \\ {0.5688} & { - 0.1759} \\ \end{array} } \right]\left[ {\begin{array}{*{20}c} {\ln y_{2,t - 4} } \\ {\ln x_{2,t - 4} } \\ \end{array} } \right] + \left[ {\begin{array}{*{20}c} { - 0.0290} \\ { - 0.0006} \\ \end{array} } \right] \\ \end{aligned} $$

15 $$ \begin{aligned} \left[ {\begin{array}{*{20}c} {\ln y_{3} } \\ {\ln x_{2} } \\ \end{array} } \right] = & \left[ {\begin{array}{*{20}c} {0.5497} & {0.2092} \\ {0.4559} & {0.3122} \\ \end{array} } \right]\left[ {\begin{array}{*{20}c} {\ln y_{3,t - 1} } \\ {\ln x_{2,t - 1} } \\ \end{array} } \right] + \left[ {\begin{array}{*{20}c} {0.1900} & { - 0.5487} \\ {0.6143} & { - 0.1879} \\ \end{array} } \right]\left[ {\begin{array}{*{20}c} {\ln y_{3,t - 2} } \\ {\ln x_{2,t - 2} } \\ \end{array} } \right] \\ & + \left[ {\begin{array}{*{20}c} { - 0.3274} & {0.1312} \\ { - 0.5366} & {0.4960} \\ \end{array} } \right]\left[ {\begin{array}{*{20}c} {\ln y_{3,t - 3} } \\ {\ln x_{2,t - 3} } \\ \end{array} } \right] + \left[ {\begin{array}{*{20}c} {0.4695} & { - 0.0421} \\ { - 0.0278} & { - 0.0851} \\ \end{array} } \right]\left[ {\begin{array}{*{20}c} {\ln y_{3,t - 4} } \\ {\ln x_{2,t - 4} } \\ \end{array} } \right] + \left[ {\begin{array}{*{20}c} { - 0.1078} \\ { - 0.0642} \\ \end{array} } \right] \\ \end{aligned} $$

16 $$ \left[ {\begin{array}{*{20}c} {\ln y_{4} } \\ {\ln x_{2} } \\ \end{array} } \right] = \left[ {\begin{array}{*{20}c} { - 0.0823} & { - 2.1360} \\ { - 0.0792} & {1.0594} \\ \end{array} } \right]\left[ {\begin{array}{*{20}c} {\ln y_{4,t - 1} } \\ {\ln x_{2,t - 1} } \\ \end{array} } \right] + \left[ {\begin{array}{*{20}c} {0.0148} & {2.007} \\ { - 0.0566} & { - 0.1254} \\ \end{array} } \right]\left[ {\begin{array}{*{20}c} {\ln y_{4,t - 2} } \\ {\ln x_{2,t - 2} } \\ \end{array} } \right] + \left[ {\begin{array}{*{20}c} {0.0001} \\ { - 0.0032} \\ \end{array} } \right] $$

### Impulse response and variance decomposition analysis results

From the impulse response and variance decomposition results, the interactive coupling of population, economy and ecological geological environment in Chengdu–Chongqing region has the conditions for emergence generation.

#### Population and economy

High-quality economic development in Chengdu–Chongqing region has a fairly long-term positive impact on sustainable population development; while sustainable population development will take up a large amount of economic resources, thus having a longer-term negative impact on high-quality economic development, so high-quality economic development requires a camera choice between investment and consumption. Economic development is the most influential factor of population development, reaching a contribution of 72% in the tenth period; while population development is only one of the elements of economic development, with a higher contribution initially, reaching 15.45% in the first period, but then begins to gradually decline to 4.79% in the tenth period.

#### Population, economy and resource elements

The population and economic development in the Chengdu–Chongqing region had a strong coercive effect on the ecological and geological environment at the beginning of this century, which was manifested by the excessive use and consumption of resource elements (arable land and water) by urban expansion at this stage. Along with the overall improvement of sustainable population development and high-quality economic development in the Chengdu–Chongqing region, the supporting role of resource elements gradually diminishes and reaches a relatively balanced state, tending to be synergistic. The contribution of resource elements to both population and economic development in Chengdu–Chongqing region is high, over 60% to population in the eighth period and over 32% to economy in the fifth period; the contribution of population and economic development to resource elements is high, with population stabilizing above 29% in the fifth period and economy stabilizing above 20% in the second period.

#### Population, economy and ecological elements

The overall coercive effect of population and economic development on ecological elements in Chengdu–Chongqing region is not strong, where the negative effect on ecological elements in the process of sustainable population evolution is slightly stronger than that of high-quality economic development. Ecological elements play a weaker but long-term positive effect on sustainable population development and high-quality economic development. The contribution of ecological elements to both population and economic development in the Chengdu–Chongqing region is low, with the population contribution to ecology stabilizing at 15% in the ninth period and the ecological contribution to population at 7% in the eighth period; the economic contribution to ecology stabilizes at 12% in the third period and the ecological contribution to economy stabilizes at 22% in the sixth period, and mainly acts in the middle and late stages. The evaluation of ecological elements mainly depends on the government's ecological protection policy and territorial policy and is not directly related to the population and economic system significantly.

#### Population, economy and ecological pressure

The Chengdu–Chongqing region first underwent an environmental policy and technology adjustment during the study period, which led to a more significant improvement in ecological pressure and a substantial reduction in the level of environmental pollution, but with the evolution of the development of the population-economic complex system, the total amount of environmental pollution increased and ecological pressure increased. In the long run, environmental pollution is continuously reduced with the improvement of the level of sustainable population development and high-quality economic development. The contribution of ecological pressure to population and economic development in Chengdu–Chongqing region is small, stabilizing at 15% for population in the 15th period and reaching 9% oscillation adjustment for economy in the 3rd period; the contribution of population development to ecological pressure is small, stabilizing at 15% in the 16th period; the contribution of economic development to ecological pressure is large, exceeding 69% in the 2nd period and tending to be stable. It shows that with the evolution of economic and social development, the intensity of economic activities has a more significant impact on environmental pollution.

#### Population, economy and ecological response to change

In the short term, population development in the Chengdu–Chongqing region will increase the government's ecological response input, while economic development will do the opposite; in the long term, population and economic development will lead to the government's input in ecological response will remain stable, with little change in input variation. In the long run, population and economic development will lead the government to invest in ecological response in a stable manner, with little change in input. The contribution of ecological response change to population development in Chengdu–Chongqing region is high, stable at more than 80% in the tenth period, and the contribution to economic development is low, slowly increasing to 7.06% in the tenth period and stabilizing; the contribution of population development to ecological response change is low, stable at 23% in the seventh period; the contribution of economic development to ecological response change is large, stable at 44% in the fifth period. It shows that economic development will influence the ecological response input more, and then act on the population development, and with the evolution of economic and social development, the ecological response will be maintained in a stable range to maintain a good ecological environment without large exogenous shocks.

In summary, there are several effects stimulated by the interaction between population and economy and ecological and geological environment in the Chengdu–Chongqing region, which lead to the transition from low to high level of the system as a whole and then generate new structures and functions, i.e., they are coupled with each other and have the conditions for emergent generation, and the generation process is characterized by time-series changes. Therefore, the thesis of coordinated and even synergistic development of population-economy-ecology-geology-environment system discussed in this paper is realistic.

## Coupling-coordinated development measurement results and analysis

On the basis of the coordination indicators in Table 1, the data are processed by using the CRITIC method, comprehensive index evaluation method and coupling coordination model^19,20^, while the comprehensive development indicators and coupling coordination degree of population, economy and eco-geological environment systems of 44 research units in Chengdu–Chongqing twin-city Economic Circle in 2000, 2010 and 2020 are calculated.

### Index analysis of population economy and ecological geological environment subsystem

The index of subsystem can be divided into 5 categories: 0–0.2 as low level, 0.2–0.4 as relatively low level, 0.4–0.6 as general level, 0.6–0.8 as relatively high level, and 0.8–1 as high level.

#### Population subsystem

**Figure 3:**
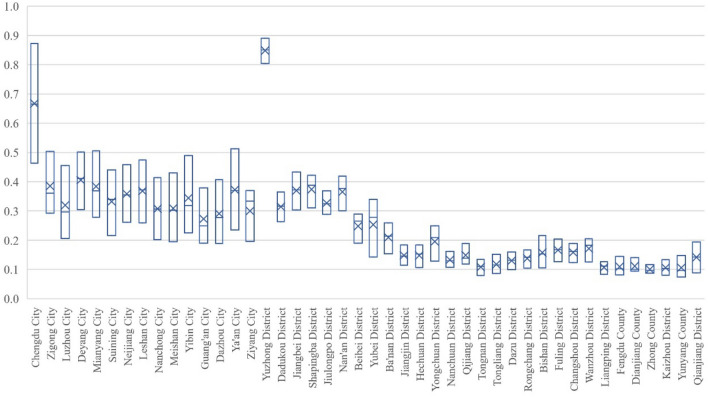
Evolution of PSI of Chengdu–Chongqing Region in 2000, 2010 and 2020.

As can be seen from Fig. 3, the PSI in Sichuan performed better than that in Chongqing from 2000 to 2020. The PSI of 4 cities in Sichuan and 24 districts in Chongqing were of low-level type in 2000; the PSI of Dazhou City in Sichuan Province was the lowest, 0.1889, and that of Chengdu City was the highest, 0.4632, while Yunyang County in Chongqing had the lowest PSI (0.0747) and Yuzhong District had the highest index (0.8039). In 2010, the PSI of 19 districts in Chongqing were still of low-level type. The PSI of Guang'an City in Sichuan province was the lowest, being 0.2488, and that of Chengdu City was the highest, being 0.6659. In contrast, the lowest PSI of Chongqing part was 0.1167 in Yunyang County and the highest was 0.8504 in Yuzhong District. In 2020, the PSI of 12 cities in Sichuan and 3 districts reached general level. The PSI of Ziyang City in Sichuan Province was the lowest, at 0.3702, and the index of Chengdu was the highest, at 0.8730, while at Chongqing part, Zhong County had the lowest index (0.1170) and Yuzhong had the highest index (0.8909). Overall, from 2000 to 2020, the PSI of Sichuan region grew the fastest in Chengdu, with an increase of 0.4098, as well as the slowest growth in Ziyang with an increase of 0.1739, while that of Chongqing had the fastest growth rate in Yubei District, with an increase of 0.1970, and the slowest growth rate in Zhong County, with an increase of 0.0294. The huge difference in PSI between Sichuan and Chongqing is mainly due to the institutional impacts of different administrative levels in the two regions.

#### Economy subsystem

**Figure 4:**
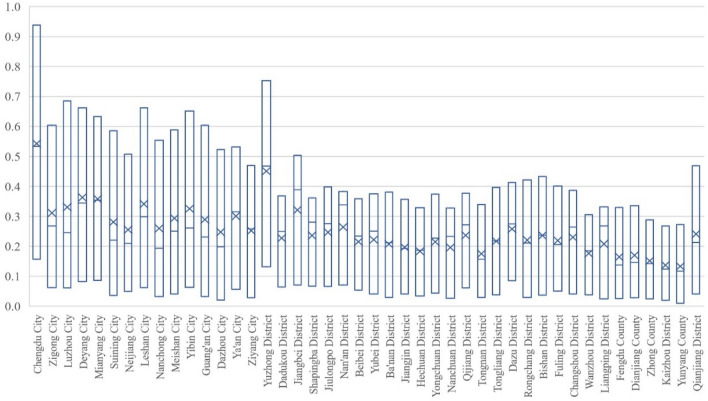
Evolution of EHI of Chengdu–Chongqing Region in 2000, 2010 and 2020.

It can be seen from Fig. 4 that all research units in Chengdu–Chongqing region were of low-level type in 2000 and the overall economic development level was not high. In 2010, the EHI of Nanchong, Dazhou in Sichuan and 9 districts in Chongqing still belonged to low-level regions, while the other regions had obvious changes, among which Chengdu and Yuzhong, as the core regions, experienced rapid economic development and reached the general level. In 2020, all regions in Chengdu- Chongqing region were out of the low-level stage, but there were significant differences between Sichuan and Chongqing: Chengdu in Sichuan entered the high-level stage, as well as Zigong, Luzhou, Deyang, Mianyang, Leshan, Yibin, Guang'an initially entered the relatively high level, while the rest cities were in the general level. However, at Chongqing part, only Yuzhong had reached the relatively high level, and Jiangbei, Dazu, Rongchang, Bishan, Fuling and Qianjiang had reached the general level, while the rest were still at the relatively low level. In summary, between 2000 and 2020, the fastest-growing economic development level of Sichuan was in Chengdu, with an increase of 0.7814, jumping from the low level to the high level, while the slowest growth was in Ziyang, with an increase of 0.4418, which also went from the low level to the general level. At the Chongqing part, Yuzhong District had the fastest-growing economic development level, with an increase of 0.6212, which went from the low level to the relatively high level, while the slowest growth was in Kaizhou District, with an increase of 0.2482, which went from the low level to the relatively low level.

#### Ecological geological environment subsystem

**Figure 5:**
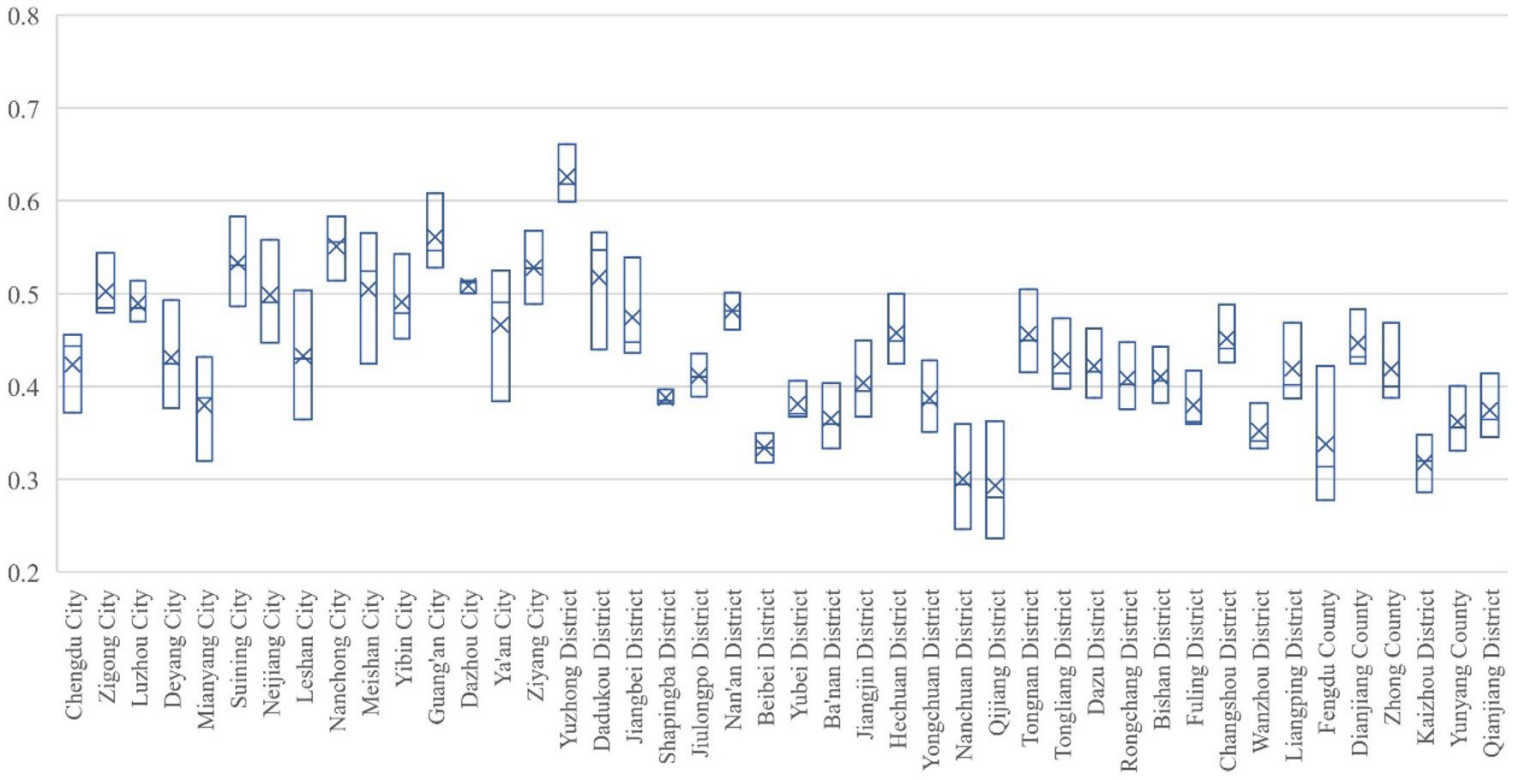
Evolution of EEBI of Chengdu–Chongqing Region in 2000, 2010 and 2020.

The evolution of EEBI in Chengdu–Chongqing region is shown in Fig. 5. In general, there are regional differences in the values and changes of the EEBI in Chengdu- Chongqing region. In 2000, the EEBI of Mianyang in Sichuan was the lowest (0.3197), while Guang'an was the highest (0.5280). At Chongqing part, the lowest EEBI occurred at Qijiang (0.2363), and the highest one was Yuzhong (0.5989). Then in 2010, Mianyang had the lowest value (0.3879) and Nanchong had the highest value (0.5557) in Sichuan, while the lowest value was 0.2803 in Qijiang and the highest value was 0.6179 in Yuzhong at Chongqing part. Time arrived at the year 2020, Mianyang was the lowest, 0.4321, while Guang'an was the highest, 0.6084. And in Chongqing, the lowest EEBI was 0.3478 in Kaizhou and the highest EEBI was 0.6607 in Yuzhong. Generally, from 2000 to 2020, the EEBI in Sichuan increased fastest in Meishan with an increase of 0.1411, and the slowest in Dazhou with an increase of 0.0136. The fastest-growing part of Chongqing was Qijiang, with an increase of 0.1260%. However, the only retrogressive area was Dadukou, with a retrogression of 0.1072%. Meanwhile, it should be noted that Shapingba had the highest score in 2010, while Wanzhou, Liangping, Kaizhou, Yunyang, Qianjiang and other places experienced the phenomenon of falling first and then rising.

#### System as a whole

The subsystems in Chengdu–Chongqing region have an upward trend, but there are significant differences in the change trend between subsystems in Sichuan and Chongqing. Judging from the overall growth rate, most cities in Sichuan showed economy >  > population > eco-geological environment, while most districts (counties) in Chongqing showed economy > eco-geological environment ~ population. And the difference between the two regions is mainly due to Chongqing, as a municipality directly under the central government, has a stronger correlation in the aspects of system, policy and administration, as well as all production factors in the region tend to be concentrated in the main urban areas, especially in Yuzhong District, which results in the population subsystem in the marginal areas (counties) developing at a slower speed than that in Sichuan. Overall, in terms of the change situation, the growth rate of the PSI in 2010–2020 was higher than that in 2000–2010 in areas far away from the core area, while the growth rate in 2010–2020 in areas close to the core area was equal to or lower than that in 2000–2010; The EHI showed that the growth rate in 2010–2020 was higher than that in 2000–2010; The EEBI showed irregular changes, which mainly depended on the ecological background, industrial structure and urban development of each place. Due to the impossibility of a significant increase in the EEBI, is unlikely to increase significantly, Thus, by 2020, for regions with faster economic development, it has started to be lower than the economic index and the population index, as well as the constraint situation is gradually emerging^21,22^.

### Spatial and temporal analysis of the coupling-coordinated development of population economy and ecological geological environment in Chengdu–Chongqing Region

#### Sichuan part

**Figure 6:**
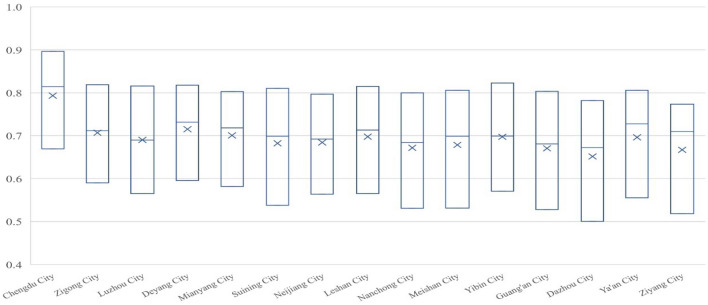
Evolution in Coupling Coordination Degree of Population Economy and Eco-geological Environment System in Sichuan from 2000 to 2020.

As shown in Fig. 6, the population economy and eco-geological environment system of 15 prefecture-level cities in Sichuan is developing in a coordinated way with a relatively good overall situation. In 2000, except for Chengdu (0.6693), which was in the stage of primary coordinated development, the other 14 cities were in the stage of reluctantly coordinated development: Zigong (0.5901), Luzhou (0.5652), Deyang (0.5958), Mianyang (0.5819), Suining (0.5380), Neijiang (0.5640), Leshan (0.5653), Nanchong (0.5311), Meishan (0.5312), Yibin (0.5708), Guang'an (0.5277), Dazhou (0.5003), Ya'an (0.5555) and Ziyang (0.5183). By 2020, 15 cities in Sichuan had reached a coordinated state, among which 11 cities were in the stage of good coordinated development: Chengdu (0.8962) was in the first place, followed by Zigong (0.8188), Luzhou (0.8159), Deyang (0.8177), Mianyang (0.8026), Suining (0.8101), Leshan (0.8146), Meishan (0.8058), Yibin (0.8229), Guang’an (0.8034), Ya'an (0.8058); Four cities were in the intermediate coordinated development stage: Neijiang (0.7969), Nanchong (0.7996), Dazhou (0.7820) and Ziyang (0.7732).

Judging from the growth amplitude, the range of the coupling coordination degree of population economy and eco-geological environment in Chongqing is between 0.20 and 0.30, as shown in Table 3.

**Table 3:** The growth amplitude of the coupling coordination degree in Sichuan part from 2000 to 2020.

Growth amplitude	Area
0.2–0.25	Leshan (0.2493), Neijiang (0.2329), Zigong (0.2286), Chengdu (0.2269), Deyang (0.2219), Mianyang (0.2207)
0.25–0.30	Dazhou(0.2817), Guang'an (0.2758), Meishan (0.2746), Suining (0.2721), Nanchong (0.2685), Ziyang (0.2549), Yibin (0.2521), Luzhou (0.2508), Ya'an (0.2503)

From the perspective of evolution process, the degree of coupled and coordinated development of the population economy and eco-geological environment system in Sichuan Province was relatively rapid and uniform in various regions during the investigation period. Meanwhile, excepting Chengdu, which remained in the coordination interval, the remaining 14 cities underwent the stages of transition and coordination. Among them, in addition to Luzhou and Yibin 2000–2010 growth rate was similar to the growth rate of 2010–2020, the rest of the 13 cities in 2000–2010 growth rate was faster than the growth rate of 2010–2020. From the coupling degree examination, except for the situation that the coupling degrees of Chengdu, Deyang and Mianyang in 2020 were less than those in 2010, the cities in Sichuan rapidly increased to 0.98 or above (the growth rate from 2000 to 2010 was much higher than that from 2010 to 2020), indicating that the economic development and ecological geological environment in all regions were highly matched in 2010 and adjusted to the dynamic coupling level in the past decade. Judging from development, all the 15 cities in Sichuan have maintained relatively rapid growth and become the main factors that promote the coupled development of the economy and ecological geological environment systems. From the original data, during the investigation period, the population of Sichuan achieved sustainable development, as well as the economy grew rapidly and the ecological geological environment was continuously improved, which made the coupling and coordination of the population economy and eco-geological environment system developed rapidly on the whole, and generally reached a relatively high level by 2020.

#### Chongqing part

**Figure 7:**
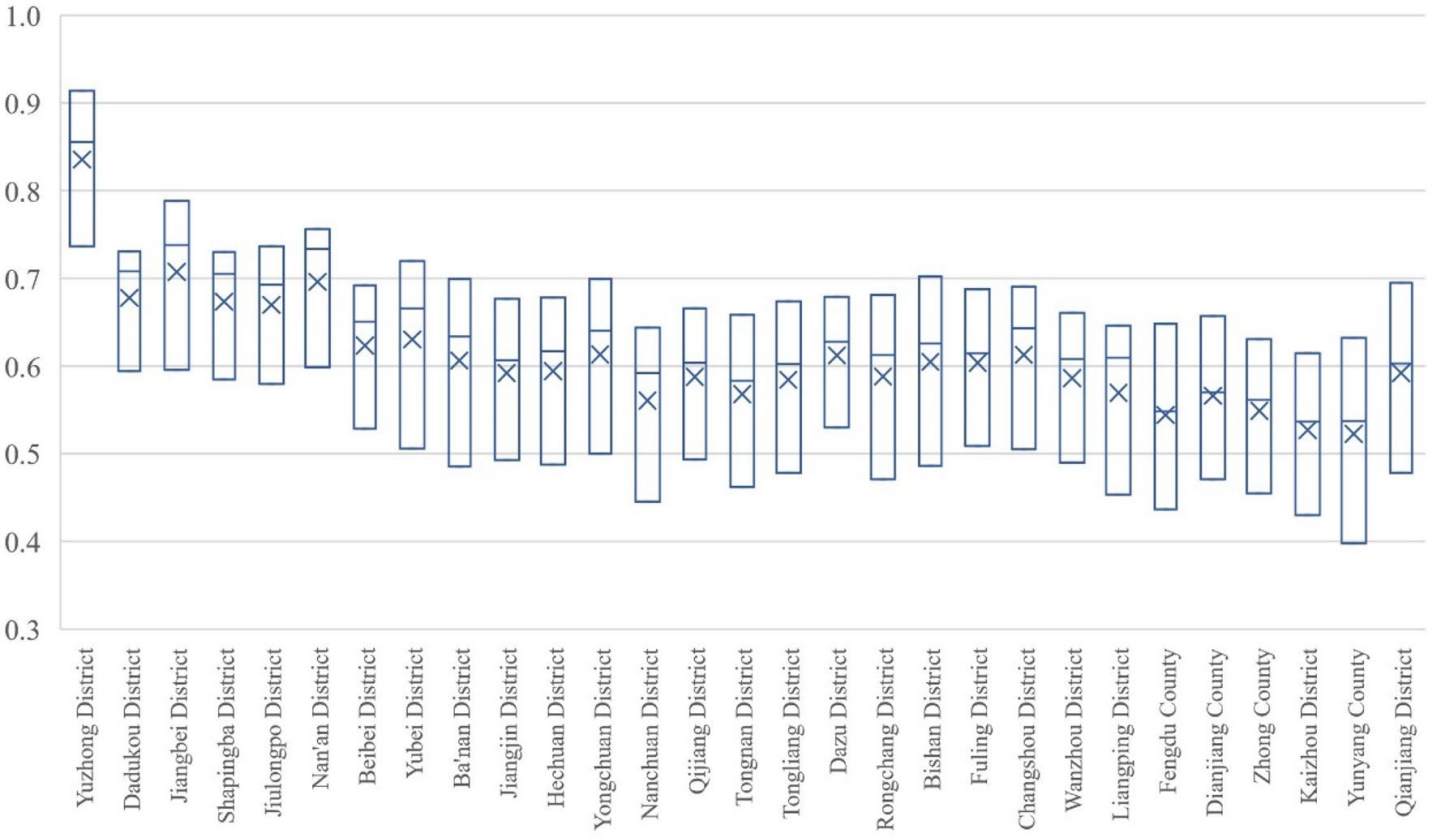
Evolution in Coupling Coordination Degree of Population Economy and Eco-geological Environment System in Chongqing from 2000 to 2020.

As shown in Fig. 7, the coupling-coordinated development of the population economy and eco-geological environment system in Chongqing shows that Yuzhong District is the best. Under its siphoning effect, the performance and growth rate in other regions generally lag behind the average level in Sichuan. In 2000, only Yuzhong District in Chongqing reached 0.7361, which was in the stage of intermediate coordinated development in the coordination zone. And there were 27 regions in transition, among which 10 regions, including Dadukou (0.5941), Jiangbei (0.5955), Shapingba (0.5842), Jiulongpo (0.5793), Nanan (0.5984), Beibei (0.5282), Yubei (0.5055), Dazu (0.5298), Fuling (0.5089) and Changshou (0.5048), were in the stage of reluctantly coordinated development, as well as Banan (0.4855), Jiangjin (0.4923), Hechuan (0.4874), Yongchuan (0.4998), Nanchuan (0.4452), Qijiang (0.4931), Tongnan (0.4931), Tongliang (0.4776), Rongchang (0.4705), Bishan (0.4857), Wanzhou (0.4897), Liangping (0.4530), Fengdu (0.4363), Dianjiang (0.4706), Zhongxian (0.4547), Kaizhou (0.4295) and Qianjiang (0.4781) were on the verge of maladjustment and antagonism; Yunyang was only 0.3978, which was in the stage of mild maladjustment antagonism and the only area in the antagonistic interval. By 2020, 29 regions in Chongqing have reached the coordination interval, among which Yuzhong District has reached 0.9136, which was at the stage of high-quality coordinated development; Dadukou (0.7302), Jiangbei (0.7883), Shapingba (0.7297), Jiulongpo (0.7366), Nanan (0.7557), Yubei (0.7195) and Bishan (0.7020) have reached the intermediate stage of coordinated development. Beibei (0.6918), Banan (0.6992), Jiangjin (0.6762), Hechuan (0.6779), Yongchuan (0.6993), Nanchuan (0.6440), Qijiang (0.6659), Tongnan (0.6579), Tongliang (0.6734), Dazu (0.6788), Rongchang (0.6808), Fuling (0.6872), Changshou (0.6903), Wanzhou (0.6606), Liangping (0.6460), Fengdu (0.6482), Dianjiang (0.6567), Zhongxian (0.6308), Kaizhou (0.6142), Yunyang (0.6321), Qianjiang (0.6947) 21 regions have reached the primary stage of coordinated development.

Judging from the growth amplitude, the range of the coupling coordination of population economy and eco-geological environment in Chongqing is larger than in Sichuan, which is between 0.10 and 0.25, as shown in Table 4.

**Table 4:** The growth amplitude of coupling coordination degree in Chongqing part from 2000 to 2020.

Growth amplitude	Area
0.10–0.15	DaDuKou (0.1361), Shapingba (0.1455), and Dazu (0.1490)
0.15–0.2	Yuzhong (0.1775), Jiangbei (0.1929), Jiulongpo (0.1573), Nanan (0.1573), Beibei (0.1636), Jiangjin (0.1840), Hechuan (0.1905), Yongchuan (0.1995), Nanchuan (0.1988), Qijiang (0.1728), Tongnan (0.1961), Tongliang (0.1958), Fuling (0.1783), Changshou (0.1855), Wanzhou (0.1709), Liangping (0.1930), Dianjiang (0.1861), Zhongxian (0. 1761), Kaizhou (0.1847)
≥ 0.2	Yubei (0.2140), Banan (0.2137), Rongchang (0.2103), Bishan (0.2163), Fengdu (0.2119), Yunyang (0.2343), and Qianjiang (0.2166)

From the perspective of evolution process, except for Fengdu and Dianjiang in Chongqing with similar growth rates in 2000–2010 and 2010–2020, the coupled and coordinated development of the system of population economy and eco-geological environment in other areas all showed a lower growth rate in 2010–2020 than in 2000–2010; In terms of the coupling degree test, each region showed rapid growth to a higher level from 2000 to 2010, while slight increase or shock adjustment from 2010 to 2020; Judging from the degree of development, each region showed a relatively uniform growth during the investigation period. According to the original data, affected by the administrative divisions, although the population of all regions in Chongqing part achieved sustainable development, the economy grew rapidly and the eco-geological environment continued to improve during the inspection period, all kinds of factors of production in some other areas of Chongqing converge to Yuzhong District, which in turn creates a unique situation in Yuzhong District, and the reason why this happened is that Yuzhong District has the optimal ecological geological environment background of rapid economic and social development and has gradually formed the competitive advantage of rapid urbanization and agglomeration economy in the process of development history. Moreover, due to the institutional differences between the municipalities directly under the central government and the provinces, the primacy effect of Yuzhong District on Chongqing is more obvious than that of Chengdu on Sichuan Province.

#### Spatial pattern analysis

**Figure 8:**
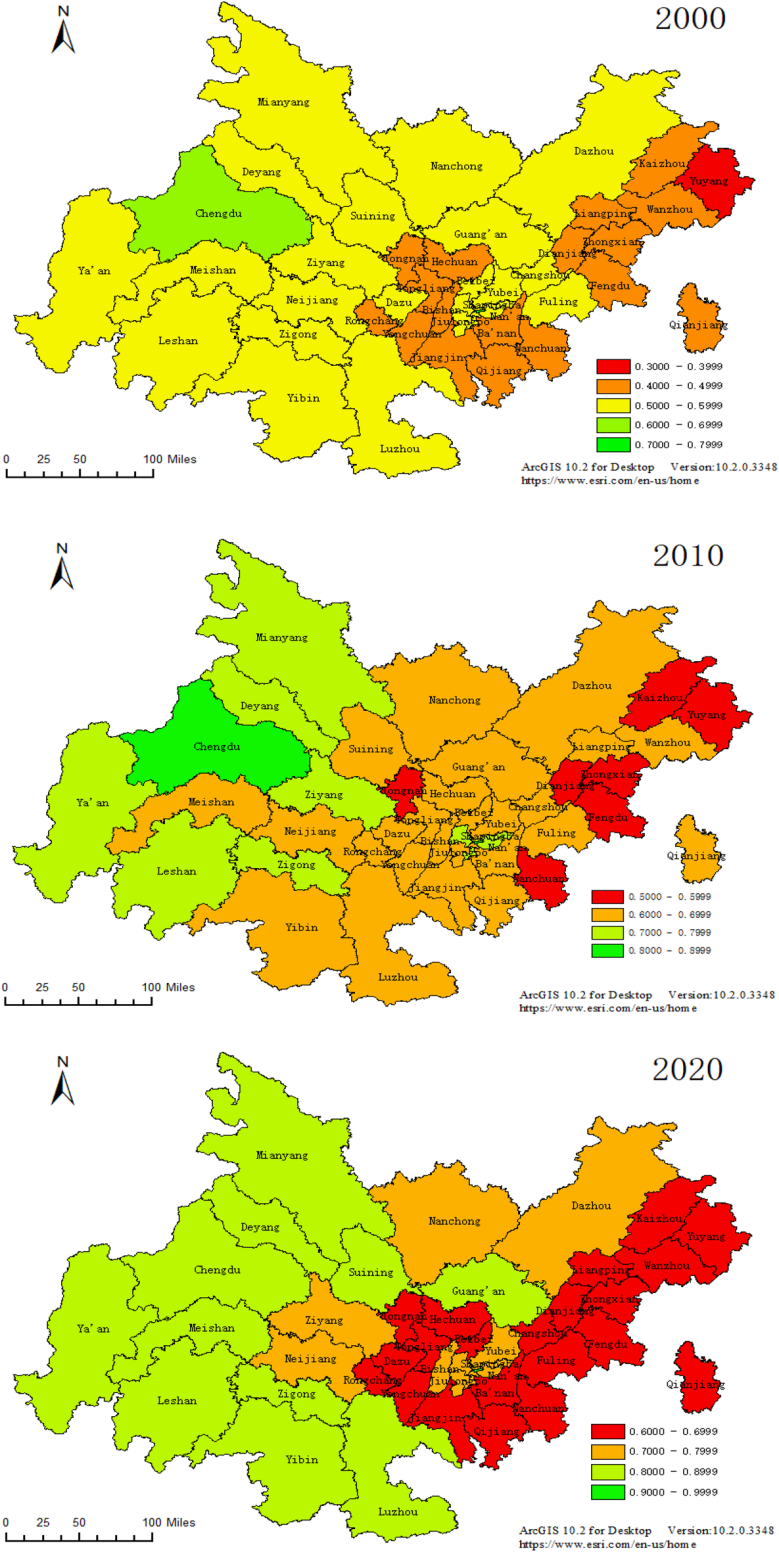
Evolution of the spatial pattern of coupled and coordinated of Population Economy and Eco-geological Environment System in Chengdu–Chongqing Region.

In order to display the spatial variation characteristics of the coupling coordination degree of the composite system in Chengdu–Chongqing region, this paper visualizes the data of 2000, 2010 and 2020 as Fig. 8. It can be seen that from the picture in 2000, the coupling-coordinated development of population, economy and ecological geological environment in Chengdu–Chongqing region was somewhat different between Sichuan and Chongqing, in which Sichuan part preceded Chongqing part in its entirety, and gradually evolving into a double helix distribution with Yuzhong District in Chongqing as the pole. Meanwhile, excepting Chengdu and Yuzhong District reached the coordination interval, the main urban area of Chongqing, Fuling-Changshou Twin-District and other cities in Sichuan were located at the upper level of the transition interval, while the former Western Chongqing, Northeastern Chongqing and Southeastern Chongqing were all located at the lower level of the transition interval and even the antagonistic interval. According to the comprehensive investigation of the original data, Chengdu and Yuzhong District were at the initial stage of industrialization to urbanization and rapid urbanization, at which time the factors of production in each region are gathering rapidly to the first city (region), resulting in the unique development pattern of Chengdu and Yuzhong District; During the 20 years of development, as the urbanization process of Chengdu gradually entered the reverse urbanization stage and the construction of characteristic urban clusters at all levels of Sichuan Province took effect, three urban agglomerations with different industrial characteristics, namely Chengdu Plain, Northeast Sichuan and Southeast Sichuan, have been gradually formed in the study area, while the coupling and coordination degree of population economy and ecological geological environment has begun to improve together.

On the other hand, compared with the coupling-coordinated development of 15 cities in Sichuan, the agglomeration effect of Yuzhong District is higher than that of Chengdu City in Sichuan Province, while this result occurs mainly since Chongqing is less independent and the factors of production are more likely to flow between regions under the restriction of administrative divisions. Even with the arrival of the reverse urbanization stage, the major urban areas in Chongqing still play the role of the second ladder of economic development and factor agglomeration, which causes the relative sluggish development of population and economy in the metropolitan area of Chongqing, Southeast Chongqing and Northeast Chongqing. Furthermore, the excellent background of the eco-geological environment in this area is restricted by the topographic and geomorphic features and the eco-environmental protection strategy, which makes the promotion of the coupling-coordinated development of population economy and eco-geological environment system slow.

### Analysis of results

Considering the relative development degree of each system, the main type of coordinated development in 2000 was “Basic synergy-Economic lag”, and there was a serious population lag problem in some areas except the main urban areas in Chongqing, while Chengdu–Chongqing region had destroyed the development of the ecological system at this time, whereas did not have a restraining effect on the population-economy system. Then, in 2010, there were differences in the main types of coordinated development. Except for Chengdu, Sichuan was mainly in “Primary synergy-Economic lag”, while Chongqing was mainly in economic lag except for Yuzhong (“Advanced synergy”) and the main urban area (“Primary synergy”). The rest of the areas were in “Basic synergy-Population lag”. Based on the policies of returning farmland to forests and concentrating industries into parks, which were implemented in the late 1990s, began to bear fruit, which made the ecological environment of Sichuan and Chongqing optimized. But it should also be noted that although the population sustainability index of the two places has been increasing year by year, the population size has been gathering to the core areas of Chengdu and Chongqing, while this phenomenon is even more obvious in Chongqing due to the continuous promotion of the policy of citizenization of rural population. At the same time, it should also be noted that Chengdu has shown the characteristics of “Ecological lag”. Then in 2020, the main types of coordinated development were “Primary synergy-Population lag”. And Chengdu, Deyang and Mianyang in Sichuan and Yuzhong in Chongqing were the core economic zones with “Advanced synergy-Ecological lag”, while the main urban areas in Chongqing were in the “Primary synergy” state of more balanced development. The regional characteristics show that, on the one hand, the social and economic development in the core area of Chengdu–Chongqing region is rapid. Although the eco-geological environment is still continuously optimized, its growth rate is lower than that of the population and economy. On the other hand, under the agglomeration of regional economy, the loss of production factors, especially the population, in the non-core area is not conducive to the sustainable development of economy and society. Aside from this, a small number of areas have achieved intermediate coordination or good coordination, indicating that the population economy and ecological geological environment have shown synchronous development characteristics at the current stage.

## Suggestions

Policy documents such as the *Outline of the Construction Plan of Chengdu–Chongqing Region Twin-City Economic Circle,* the *Joint Implementation Plan of Chongqing and Sichuan Provinces and Municipalities for Implementing* and the *Outline of the Construction Plan of Chengdu–Chongqing Region Twin-City Economic Circle* have scientifically designed and guided the development path of twin-city economic circle in Chengdu–Chongqing region. Therefore, based on the coordinated development and evolution of the system of population, economy eco-geological environment in Chengdu–Chongqing region, this paper puts forward the following three suggestions.

The first is to promote the optimization of the population structure at different levels in Chengdu–Chongqing region with the characteristics of new urbanization. On the one hand, at the macro level, the Chengdu–Chongqing-Xi’an economic growth iron triangle will be jointly constructed to develop the Chengdu–Chongqing twin-city and Xi'an city economic circle into the economic growth pole, strategic support level and transportation hub of “the belt and road initiative” and Yangtze River Economic Belt. On the other hand, at the mid-regional level, it is necessary to develop small and medium-sized cities, which will break through the urban structure of traditional industries with “characteristic urbanization”, while building livable cities with harmonious economic, social, cultural and ecological development, and forming differentiated competitive advantages with large cities. In the end, at the regional level of urban and rural areas, combined with the rural revitalization strategy, the towns and farms will be turned into “new small towns”, so as to attract the rural population and related industries.

The second is to promote the optimization and upgrading of the industrial structure in Chengdu–Chongqing region with “new infrastructure” and information technology. First of all, a national industry-academic-research center with national security as the core will be built, relying on the research system of Chengdu–Chongqing regional scientific which is originally used to focus on national defense science and technology research and development. Secondly, relying on the land location advantage of Chengdu–Chongqing region in the “the belt and road initiative” strategy, while undertaking the transfer of traditional industries in the east, the whole industrial chain should be cleaned and energy-saving, and transformed to intelligent manufacturing through the “industrial internet” to increase the added value of traditional industries and meet the product demand of ecological civilization. Then, in order to give full play to its superior ecological environment endowment and relatively low cost of resources and energy, a new strategic resource hub will be built in Chengdu–Chongqing region, while building industrial clusters such as 5G, UHV, big data center, artificial intelligence, intercity high-speed railway and rail transit.

The third is to strengthen the coordinated management of cross-border pollution and promote the construction of ecological barriers in the upper reaches of the Yangtze River. Therefore, it is necessary not only to promote regional joint governance, joint construction and protection, but also to establish a unified industry standard for environmental systems such as air, water, soil and solid waste in Chengdu and Chongqing, while building an information exchange platform to carry out the whole-process supervision and management from the source to the end. Meanwhile, explore and implement the horizontal ecological compensation transfer mechanism and carbon sink storage and trading mechanism of the Yangtze River Economic Zone and the Yellow River Economic Zone to ensure national ecological security. And it is necessary to scientifically assess the investment and comprehensive benefits of ecological environmental protection in the Chengdu–Chongqing region and set up a special fund for ecological compensation, which will be used specifically for ecological construction in the Chengdu–Chongqing region and the construction of national key ecological function areas.

## Data Availability

The datasets used and analyzed during the current study available from the corresponding author on reasonable request.
